# Litter decomposes slowly on shaded steep slope and sunny gentle slope in a typical steppe ecoregion

**DOI:** 10.1002/ece3.6933

**Published:** 2021-02-26

**Authors:** An Hu, Yaning Duan, Lei Xu, Shenghua Chang, Xianjiang Chen, Fujiang Hou

**Affiliations:** ^1^ State Key Laboratory of Grassland Agro‐Ecosystems Key Laboratory of Grassland Livestock Industry Innovation Ministry of Agriculture Lanzhou China; ^2^ College of Pastoral Agriculture Science and Technology Lanzhou University Lanzhou China

**Keywords:** aspect, carbon and nutrient cycling, slope, soil moisture, solar radiation

## Abstract

Plant litter decomposition is mainly affected by litter properties and environmental factors, but the influence of terrain on litter decomposition is not well understood. We studied the effects of terrain on litter decomposition over a period of 12 months at six locations in a typical steppe ecoregion and measured the concomitant release of carbon (C), nitrogen (N), and phosphorus (P). The study site has two aspects, shaded and sunny, each aspect having three slopes: 15°, 30°, and 45°. The same mixed litter was used at each location to exclude the influence of litter quality variation. Results showed that soil temperature and moisture, solar radiation, and plant species diversity varied by terrain, which in turn, affected the *k*‐value (standardized total effects, 0.78, 0.12, 0.92, 0.23, respectively) and the release of C (0.72, –0.25, 0.83, 0.24, respectively), N (0.89, –0.45, 0.76, 0.40, respectively) and P (0.88, 0.77, 0.58, 0.57, respectively). *K*‐value and C release decreased with increasing slope on shaded aspect, while increased with increasing slope on sunny aspect. The release of N and P decreased with increasing slope on the shaded aspect. *K*‐value and C, N, and P release were significantly higher on shaded than that on sunny aspect at 15° and 30°, while at 45°, it was higher on sunny than on shaded aspect. The litter mass loss was slower on shaded 45° and sunny 15°. So moderate grazing or mowing could be used to reduce litter accumulation and accelerate litter decomposition on these terrains. Structural equation modeling indicated that soil temperature and solar radiation had the greatest influence on *k*‐value and C, N, and P release, and these two factors were directly related to soil moisture and plant species diversity. Overall, our results emphasize the need to consider terrain for litter decomposition in typical steppe ecoregions.

## Graphical abstract

Soil temperature and moisture, solar radiation, and plant species diversity varied by terrain (i.e., slope incline and directional aspect), which in turn, affected the litter mass loss and release of carbon, nitrogen, and phosphorus.

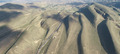

## 1 INTRODUCTION

Litter decomposition is the main process for returning vegetation biomass to terrestrial ecosystems by which organic matter is converted to simpler organic and inorganic materials, ant it serves to complete the cycles of carbon (C), nitrogen (N), and phosphorus (P) for the function and maintenance in of terrestrial ecosystems (Bornman et al., 2015; Braun et al., 2019; Petraglia et al., 2018). Litter serves as a “bridge” for connecting living plants with soil to facilitate energy and nutrient transfer (Sun et al., 2018). Thus, litter decomposition is critical for regulating the availability of soil nutrients and therefore the productivity of terrestrial ecosystems.

Many studies on the decomposition of litter are carried out on flat ground, and they have identified abiotic and biotic factors that are key drivers of litter decomposition in arid and semiarid areas (Austin et al., 2009; King et al., 2012; Santonja et al., 2018). However, the distribution of solar radiation, soil moisture and temperature, litter quality and quantity, and decomposer diversity varies with terrain. Solar radiation, soil temperature, and moisture are all significantly affected by the aspect and slope of land, whereas solar radiation is less intense on shaded than on sunny aspect and decreases with increasing slope (Arnold & Rees, 2009; Yang et al., 2007). Soil temperature and moisture, either alone or in combination, can affect the rate of litter decomposition directly by altering the activity of the decomposers or indirectly by changing the composition and abundance of plant species, thereby changing the quality and quantity of litter. Higher soil moisture and temperatures generally increase the rate of litter decomposition (Lee et al., 2014; Petraglia et al., 2018). Also, vegetation is distributed differently among different slopes and aspects and responds differently to varied amounts of solar radiation (Dagon & Schrag, 2019). In recent years, a large number of studies have shown that although the intensity, direction, and mechanism of photodegradation differ among studies, photodegradation can be an important driver of litter decomposition in arid and semiarid ecosystems (Almagro et al., 2016; Asperen et al., 2015; Austin & Vivanco, 2006).

Despite recent interest in the roles abiotic and biotic factors play as drivers of litter decomposition in drylands, it is largely unknown how terrain affects the litter decomposition by affecting the amount of incident solar radiation, soil temperature and moisture, and plant species diversity. Here, we conducted a multifactorial 12‐month litterbag decomposition experiment on six different terrains (i.e., slope incline and directional aspect) in the Loes1s Plateau, China, (a typical steppe ecoregion), to investigate how terrain influence early‐stage of litter decomposition. Carbognani et al. (2014) and Djukic et al. (2018) found that litter quality has a greater effect on early‐stage mass loss than do soil moisture and temperature. In this study, the same litter pool was used for all replicates to eliminate any influence of litter quality. Specifically, we aimed to: (a) investigate the influence of terrain on the rate of mass loss and release rates of C, N, and P; and (b) measure how solar radiation, soil temperature and moisture, and plant species diversity vary with terrain.

## 2 MATERIALS AND METHODS

### 2.1 Site description

**FIGURE 1:**
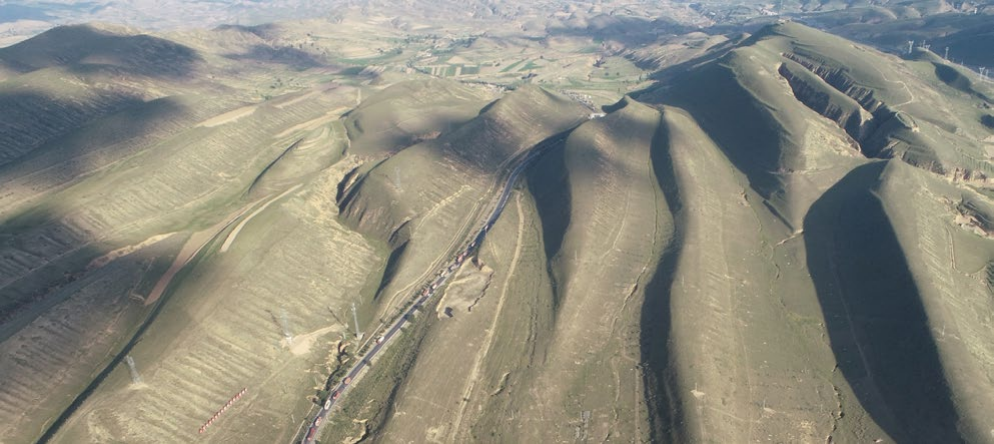
Landforms of the Loess Plateau with shaded and sunny aspect and each of them have different steep of slopes. This is a photograph of our study site, taken by Fengshuai Lu with a drone in spring 2019

The study site was in Huanxian County of Gansu Province, northwest China (37°07′N, 106°48′E), which is a pastoral farming basin with hilly terrain (Figure 1). The average elevation is 1700 m with a historical average daily temperature of 7.5°C over the 12‐month study period. The site experiences long, cold winters (frost‐free period 125 days), and hot summers (mean annually accumulated temperature is 3633 degree‐days above 0°C). The mean annual precipitation over the last 17 years is 265 mm, with 1993 mm annual evaporation. More than 70% of the annual precipitation occurs from July through September, and the annual precipitation amplitude of variation is 45%–100%, which is typical for continental monsoon climates (Hu et al., 2019). The vegetation in the study area is dominated by three herbaceous plant species: *Artemisia capillaris* Thunb., *Lespedeza davurica* (Laxm.) Schindl., and *Stipa bungeana* Trin., which together account for more than 80% of the total plant biomass in the study area (Hu et al., 2019).

### 2.2 Experimental design

#### 2.2.1 Litter collection, arrangement, and sampling

Plots were established at the study site on opposing aspects along a long hill typical of the terrain in the Loess Plateau, China. The hill and its ridges are aligned in a northwest‐southeast direction. The study assessed two parameters: slope and aspect. The slope parameter had three levels representing the pitch: 15°, 30°, and 45°. The aspect parameter had two levels: sunny, southwest‐facing aspect, and a shaded, northeast‐facing aspect. Each combination had three replicates. Each replicate land area was 15 m × 50 m and was randomly assigned to the areas representing the slope and aspect combinations.

**FIGURE 2:**
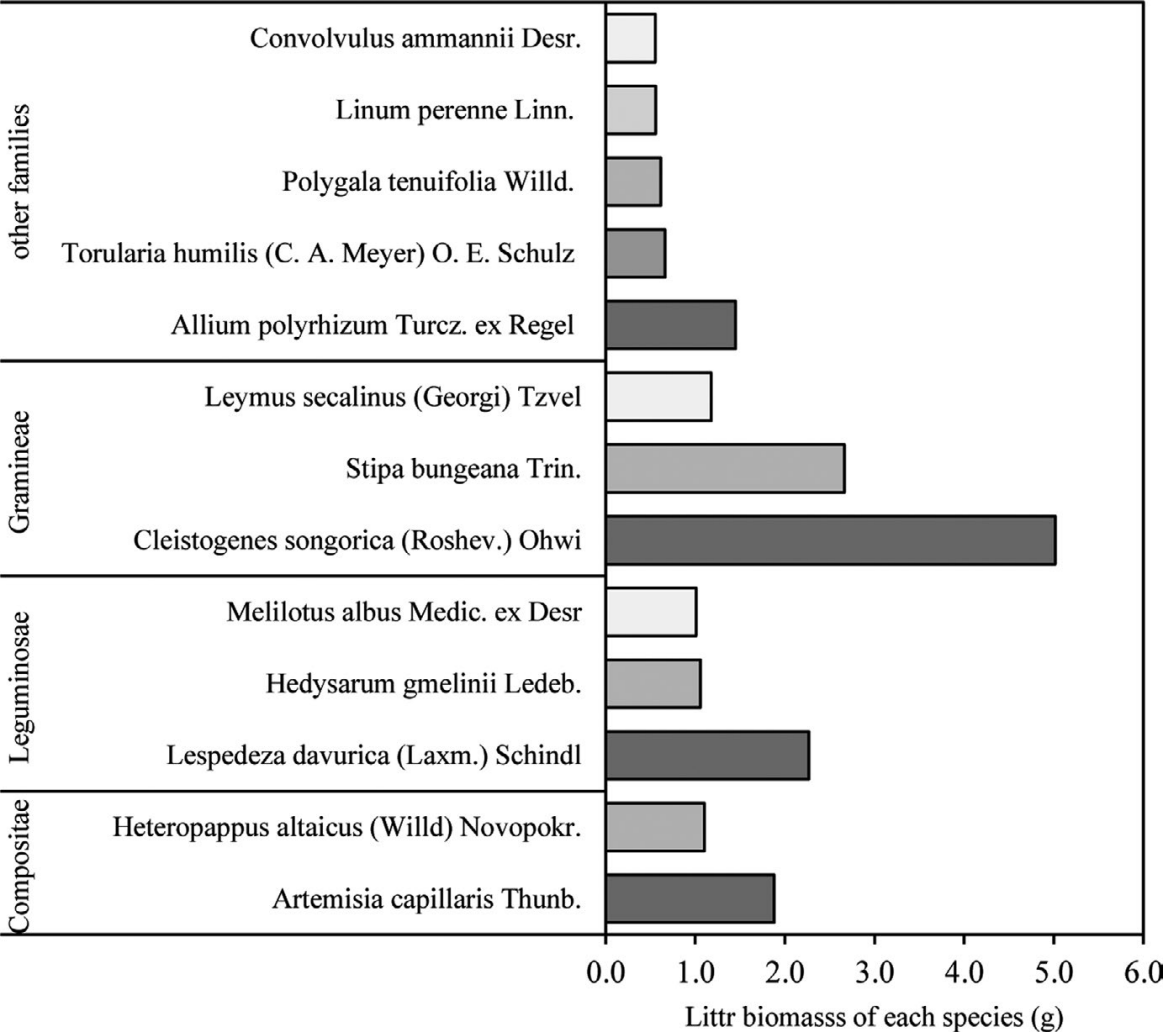
Composition of mixed litter from six terrains, and total weight of each mixed litter sample is 20 g

Initial litter biomass (g/m^2^) was estimated in November 2010 in each of the plots using quadrat methods. Within each plot, a 1 m^2^ quadrat was randomly selected and litter biomass collected within the quadrat. A total of four quadrats were sampled, and these represented subsamples within the replicate. Rocks, animal residues, seedlings, and soil were removed from the litter. The biomass was then placed in bags and taken to the laboratory where it was dried at 65°C to constant weight and weighed. All the litter samples were mixed, and then 20 g of dried material was placed into nylon net bags (size 15 cm × 25 cm, aperture 0.5 mm). The mixed litter comes from all the litter in the six terrains, including leaves and branches. These include Compositae (*Artemisia capillaris* Thunb. And *Heteropappus altaicus* (Willd) Novopokr.), Leguminosae (*Lespedeza davurica* (Laxm.) Schindl, *Hedysarum gmelinii* Ledeb., *Melilotus albus* Medic. ex Desr), Gramineae (*Cleistogenes songorica* (Roshev.) Ohwi, *Stipa bungeana* Trin. *Leymus secalinus* (Georgi) Tzvel), and other families (*Allium polyrhizum* Turcz. ex Regel, *Torularia humilis* (C. A. Meyer) O. E. Schulz, *Polygala tenuifolia* Willd., *Linum perenne* Linn., *Convolvulus ammannii* Desr.) (Figure 2). Mixed litter was used in this study to allow investigation of terrain effects without being confounded by variations in litter quality (initial litter C, N, and P). Each replicate litter sample was placed in a litter bag, with 216 bags total, representing the slope/aspect parameters, replicate, and time period combinations: three subreplicates × three replicates × two aspects × three slope positions × four sampling events. A subset of litter bags was kept representing the time zero litter condition. The remaining bags were numbered, and in April 2011, they were placed directly on the soil surface in the appropriate replicate plots and tethered to the soil surface with wire. Subsets of the remaining litterbags were removed at 5, 8, and 12 months after placement. Soil and plant materials that had accumulated on the surfaces of the bags were carefully removed. After removal from the field, litter bags were dried at 65°C to constant weight, and weights were recorded. After drying, biomass from the litter bags was crushed and sieved. An external heating‐potassium dichromate oxidation method, the Kjeldahl method, and molybdenum diatomite colorimetric method were used to assess total C, total N, and total P, respectively, for each litter sample.

Plant species diversity was used to represent the surrounding environment, as it is strongly dependent on the terrain (Gholz et al., 2000). The plant species information from the quadrats was used to calculate the Shannon–Wiener diversity index (*H*) (1):

(1) $$H=\sum_{i}^{S}p_{i}\ln p_{i},$$

where *p_i_* is the proportional weight of an individual species, *i*, of the total aboveground biomass, and S is the total number of species (Dougall & Dodd, 1997).

#### 2.2.2 Soil temperature and moisture

The surface soil moisture and temperature were measured within the top 10 cm in each replicate plot at each mid‐month throughout the study period. Soil moisture was determined gravimetrically. The soil was removed and placed in a tin and then weighed to determine the wet weight of the soil. Soil samples were then transported to the laboratory where they were oven‐dried to constant weight at 105°C to determine dry weight. Soil temperature was recorded at 8 a.m., 2 p.m., and 8 p.m. using a thermometer.

#### 2.2.3 Solar radiation

Solar radiation was recorded once every hour by a weather station at the study site. The weather station is in a flat area. Compared with the weather station, the six sampling sites differed only in slope and aspect. The solar radiation in each terrain was calculated by recorded solar radiation measurements from the weather station (Jin et al., 2014; Wang et al., 2002).

Total solar radiation (${\text{SR}}_{\text{total}}$):

(2) $${\text{SR}}_{\text{total}}={\text{SR}}_{d}+{\text{SR}}_{s}+{\text{SR}}_{r},$$

Direct solar radiation (${\text{SR}}_{d}$):

(3) $${\text{SR}}_{d}={\text{SR}}_{0}\tau_{b}\cos\theta ,$$

(4) $${\text{SR}}_{0}=S_{0}\times \left(1+0.0344\;\text{cos(}360^{\circ }N/365)\right)$$

Scattered radiation (${\text{SR}}_{s}$):

(5) $${\text{SR}}_{s}={\text{SR}}_{0}\times \left(0.271‐0.294\tau_{b}\right)\times \cos^{2}\left(\text{slope}\right)/2\sin\;\text{aspect,}$$

Reflection radiation (${\text{SR}}_{r}$):

(6) $${\text{SR}}_{r}=\rho \times {\text{SR}}_{0}\times \left(0.271+0.706\tau_{b}\right)\times \sin^{2}\left(\text{slope}\right)/2\sin\;\text{aspect,}$$

where ρ is the surface reflectance, ${\text{SR}}_{0}$ is solar radiation intensity incident to the upper boundary of the atmosphere, *N* is the number of days in one year, and $S_{0}$ is the solar constant (the amount of solar radiation per unit area of the upper boundary of the atmosphere perpendicular to the direction of solar irradiation), which was taken as 1367 W/m. The coefficient of atmospheric transparency ($\tau_{b}$) was same at six terrains in this study, and θ is the angle of optical incidence on an inclined surface under certain topographic conditions; α is the altitude angle of the sun.

### 2.3 Data analysis

The decomposition rate (*k*, g 20 g^–1^ day^–1^) of litter mass was assessed using a negative exponential model (Swift et al., 1979) (7):

(7) $$\frac{x_{t}}{x_{0}}=e^{‐(t\times k)},$$

The release rate of nutrients (C, N, and P) was calculated by:

(8) $$r_{i}=\frac{x_{0}\times Pi_{0}‐x_{t}\times Pt_{0}}{t},$$

where $x_{0}$ is the initial litter dry mass, $x_{t}$ is the dry litter mass at time *t* (days), $r_{i}$ is the release rate of nutrients, and $Pi_{0}$ and $Pt_{0}$ represent the total nutrient concentration at the initial time and at a later time *t*, respectively.

**FIGURE 3:**
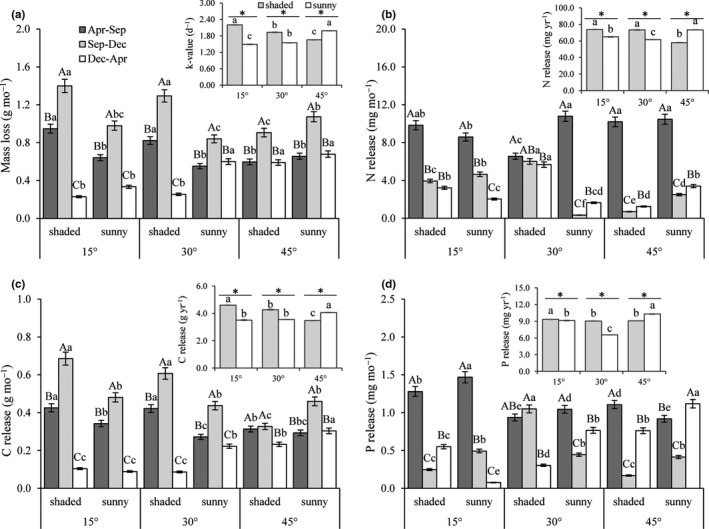
Monthly and annual rates of litter mass decomposition and release rates of C, N, and P in two aspects (shaded and sunny) with three slopes (15°, 30°, and 45°) in different sampling periods. In each histogram, different lowercase letters indicate significant differences among different terrains at same decomposition period (*p*‐value < .05); different capital letters indicate significant difference among different decomposition periods at some terrain (*p*‐value < .05). In the upper right corner of each histogram, different lowercase letters indicate significant differences among slopes at each aspect (*p*‐value < .05), and within each slope, an asterisk indicates a significant difference between the shaded and sunny aspect (*p*‐value < .05)

The soil temperature and moisture, solar radiation, plant species diversity, and litter mass loss and C, N, and P release data were subjected to a goodness‐of‐fit test (Shapiro–Wilk test). The results indicated that the data collected from this study were normally distributed. To test for differences in soil temperature and moisture, solar radiation, and plant species diversity (Table 2) and annual rate of litter mass decomposition and C, N, and P release (Table 3), a general linear model was applied, with slope, aspect, and their interactions as independent factors. And they were verified by Tukey's post hoc test. One‐way ANOVA was used to compare the difference of monthly litter mass loss and C, N, and P release among different decomposition periods and terrains (Figure 3). One‐way ANOVA also used to compare the difference of annul decomposition rate of litter mass and release rates of C, N, and P (in the upper right corner of each histogram of Figure 3) among different slopes and aspects. All analyses were carried out using SAS 9.3 software (SAS Institute Inc.). Only the data from the final litter bag collection (i.e., 12 months) were used in the general linear model and post hoc test for difference analyses.

Structural equation modeling (SEM) was used to estimate the contributions of slope and aspect (shaded, 1; sunny, 2) to responses of the soil (moisture and temperature), solar radiation, and species diversity, as well as their effects on rate of litter mass decomposition (*k*‐value) and the annual release of C, N, and P. The main advantage of SEM is that it can assess complex causal relationships between variables by translating hypothetical causal relationships into expected statistical relationships in the data (Doncaster, 2007). We assumed that slope steepness and aspect had the potential to directly alter the k‐value and release rates of C, N, and P as well as indirectly through changes in the soil (moisture and temperature), solar radiation, and plant species diversity in the model. The chi‐square test was used to judge the fit of the SEM. For the analyses, a good fit of the mode is when 0 ≤ χ^2^/*df* ≤ 2 and 0.05 < *p‐*value ≤ 1. SEM was carried out using AMOS 21 software (Arbuckle, 2010).

## 3 RESULTS

### 3.1 Terrain effect on plant species diversity and abiotic parameters

The plant species diversity was greatest on the 30° shaded slope, with a value of 2.03, and decreased with increasing slope on the sunny aspect (Table 1). Slope, aspect, and their interaction all had significant effects on plant species diversity (Table 2).

**TABLE 1:** Annual mean soil moisture and temperature, annual total solar radiation, and plant species diversity of two aspects (shaded and sunny) with three slopes angles (15°, 30°, and 45°)

Aspect	Slope	Soil moisture (%)	Soil temperature (°C)	Solar radiation (MJ/m^2^ year^−1^)	Plant species diversity
Shaded	15°	6.5 ± 0.2c	9.4 ± 0.1c	5951.9 ± 8.6bc	1.51 ± 0.07c
	30°	6.6 ± 0.1c	9.2 ± 0.1c	5907.2 ± 11.3c	2.03 ± 0.22a
	45°	9.4 ± 0.2a	8.6 ± 0.1d	5721.8 ± 13.4d	1.50 ± 0.05c
Sunny	15°	6.5 ± 0.2c	11.4 ± 0.2b	5977.3 ± 8.2b	1.64 ± 0.13 b
	30°	6.9 ± 0.1c	11.6 ± 0.1b	6013.4 ± 15.3ab	1.40 ± 0.05c
	45°	8.3 ± 0.1b	12.5 ± 0.2a	6091.9 ± 19.1a	1.36 ± 0.07c

Note

Data presented are mean values ± standard error. Different letters indicate significant differences among the six kinds of terrain (*p*‐value < .05).

**TABLE 2:** Effects of slope (15°, 30°, 45°) and aspect (shaded and sunny) on annual mean soil moisture and temperature, solar radiation, and plant species diversity

Parameter	*df*	Soil moisture (%)		Soil temperature (°C)		Solar radiation (MJ/m^2^ year^−1^)		Plant species diversity	
		*F*	*p*‐Value	*F*	*p*‐Value	*F*	*p*‐Value	*F*	*p*‐Value
Slope	2	167.02	<.001	1.33	.301	160.40	<.001	10.34	.007
Aspect	1	5.40	.038	955.55	<.001	7.92	.006	5.89	.017
Slope × aspect	2	11.69	.002	42.87	<.0001	62.18	<.001	11.22	.002

Slope, aspect, and their interaction all had significant effects on soil moisture (Table 2). Both aspect and the interaction between aspect and slope significantly affected soil temperature, although slope alone did not (Table 2). Soil moisture increased with increasing slope on both shaded and sunny aspect (Table 1). At 15° and 30° slopes, there was no significant difference in soil moisture between the shaded and sunny aspect, but the soil moisture of the shaded 45° (9.4%) was significantly greater than that of the sunny 45° (8.3%) (Table 1). For the shaded aspect, soil temperature decreased with increasing slopes; for the sunny aspect, however, soil temperature increased with increasing slopes. Soil temperature was higher on sunny aspect than on shaded aspect (Table 1).

Slope, aspect, and their interaction all had significant effects on solar radiation (Table 2). For the shaded aspect, solar radiation decreased with increasing slopes; for the sunny aspect, however, solar radiation increased with increasing slopes. Solar radiation was higher on sunny than on shaded aspect (Table 1).

### 3.2 Monthly and annual decomposition rate of litter mass

Monthly litter mass loss was the fastest in the second decomposition period (Sep to Dec) at each terrain. On 45° slopes, the litter mass loss on sunny aspect was always faster than that on shaded aspect in all three decomposition periods (Figure 3a).

Annual decomposition of litter mass was estimated using a decay coefficient (*k*‐value; Figure 3a). Slope and aspect, as well as their interaction, significantly affected the *k*‐value (Table 3). On sunny aspect, the k‐value increased with increasing slope, that is, 1.45 at 15°, 1.54 at 30°, and 1.99 at 45°. However, it decreased with increasing slope, that is, 2.20 at 15°, 1.93 at 30°, and 1.66 at 45° on shaded aspect. On both the 15° and 30° slopes, *k*‐values for the shaded aspect were significantly higher than on sunny aspect, but at 45° slopes, it was significantly higher on the sunny compared to the shaded aspect (*p*‐value < .01; Figure 3a).

**TABLE 3:** Effects of slope (15°, 30°, 45°) and aspect (shaded and sunny) on annual decomposition of litter mass (*k*‐value) and release rate of C, N, and P

Parameter	*df*	*k*‐Value		C (g/year)		N (mg/year)		P (mg/year)	
		*F*	*p*‐Value	*F*	*p*‐Value	*F*	*p*‐Value	*F*	*p*‐Value
Slope	2	211.75	<.001	104.50	<.001	79.01	<.001	1888.39	<.001
Aspect	1	53.03	<.001	653.44	<.001	56.11	<.001	359.69	<.001
Slope × aspect	2	443.54	<.001	1013.22	<.001	1294.74	<.001	1697.37	<.001

### 3.3 Monthly and annual litter release rate of litter nutrient

Monthly release rate of litter C was the fastest in the second release period (Sep to Dec) at each terrain. In first release period (Apr to Sep), litter C release was faster on shaded compared to sunny aspect at each slope. (Figure 3b).

Litter annual C release rate was affected by slope and aspect, as well as their interaction (Table 3). On sunny aspect, the annual C release rate increased with increasing slope, and it was significantly higher for 45° slopes (4.06 g/year). On shaded aspect, the annual C release rate significantly decreased with increasing slope, that is, 4.60 g/year at 15°, 4.27 at 30°, and 3.48 at 45°. For both the 15° and 30° slopes, the annual C release rates on the shaded slopes were significantly higher than on sunny slopes; however, it was significantly higher on sunny slopes than on shaded slopes at 45° (*p*‐value < .01; Figure 3b).

Monthly release rate of litter N was faster in first release period in each terrain. It was faster on sunny than on shaded aspect at 45° in all three periods. At 30°, litter N monthly release was faster on shaded than on sunny aspects in first release period (Apr to Sep), while in the second and third periods, it was faster on shaded compared with sunny aspect (Figure 3c).

Annual release rate of litter N decreased with increasing slope on shaded aspect, with 73.84 mg/year at 15°, 73.47 at 30°, and 58.02 at 45°. On sunny aspect, the annual N release rate was significantly higher for the 45° slopes, with 73.42 mg/year, than for the 30° (61.52 mg/year) or 15° (64.99 mg/year; Figure 3c) slopes. Slope and aspect, as well as their interaction, significantly affected the litter annual N release rate (Table 3). The annual N release rates on the shaded aspect were significantly higher than on the sunny aspect at 15° and 30°. However, it was significantly lower on shaded aspect than on sunny aspect at 45°.

Slope and aspect, as well as their interaction significant affected annual P release (Table 3). The annual release rate of litter P was significantly higher on 15° (9.34 mg/year) than on 30° and 45° slopes in shaded aspect (Figure 3d). On sunny aspect, the annual release of P was significantly higher on 45° (10.30 mg/year) than the 30° (6.55 mg/year) and 15° (9.08 mg/year) slopes. Annual P release was significantly higher on the shaded than on the sunny aspect for the 15° and 30° slopes. This trend was reversed for the 45° slope, with significantly higher annual P release on sunny aspect (Figure 3d).

### 3.4 Structural equation modeling for *k*‐value and C, N and P release

**FIGURE 4:**
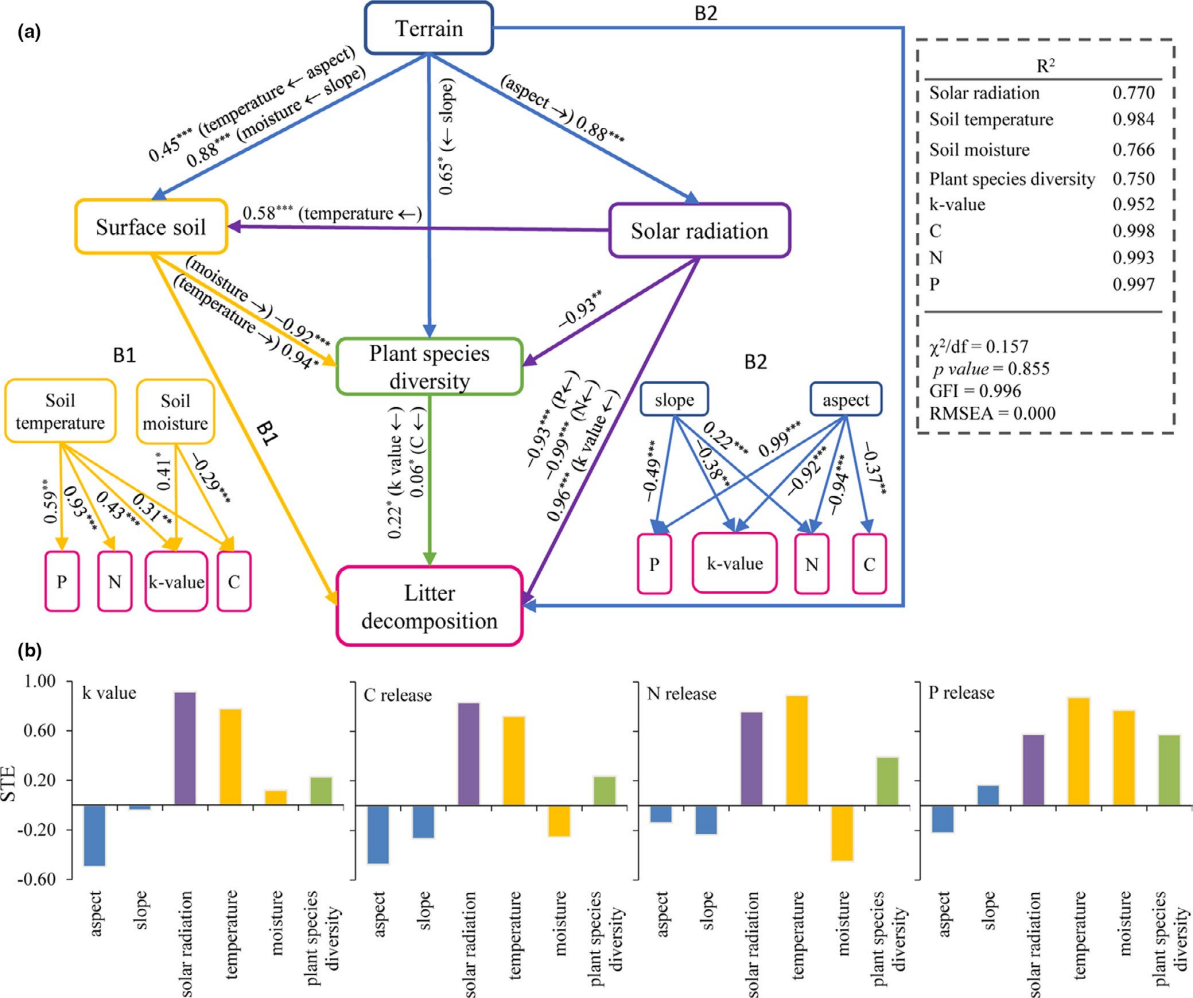
(a) Structural equation modeling for slope and aspect based on the data for surface soil temperature and moisture, solar radiation, and plant species diversity. The lines indicate significant direct effects. The numbers along the arrows are the standardized path coefficients, indicating the strength of the relationships. The *R* ^2^ value represents the proportion of total variance for the dependent variable of interest. Significance levels of the direct effects are represented as: *p‐value < .05, **p‐value < .01, ***p‐value < .001. (b) Standardized total effects (STE) from the SEM; the sum of direct and indirect effects from each variable on *k*‐value and C, N, and P release rates

The SEM was used to describe the influence of terrain on the decomposition of litter. The results of the SEM indicated that slopes have significant positive direct effects on soil moisture and plant species diversity with a standardized path coefficient (SPC) of 0.88 (*p*‐value < .001) and 0.65 (*p*‐value < .05), respectively. Aspect was found to have significant positive direct effects on soil temperature and solar radiation with SPCs of 0.45 (*p*‐value < .001) and 0.88 (*p*‐value < .001), respectively. Slope and aspect both had negative direct effects on the *k*‐value with SPCs of −0.38 (*p*‐value < .01) and −0.92 (*p*‐value < .001), respectively). For C release, only aspect had a negative effect (SPC −0.38, *p*‐value < .01). Aspect had a negative effect on N release (SPC −0.94, *p*‐value < .001), whereas slope had a positive effect on N release (SPC 0.22, *p*‐value < .001). For P release, slope and aspect had opposite effects with SPCs of −0.49 and 0.99 (both *p*‐value < .001), respectively. Solar radiation had positive effects on soil temperature and *k*‐value, with SPCs of 0.58 and 0.96 (both *p*‐value < .001), respectively, and negative effects on the plant species diversity and release rate of N and P with SPCs of −0.93 (*p*‐value < .01), −0.93 (*p*‐value < .001), and −0.99 (*p*‐value < .001), respectively. Plant species diversity had a positive effect on *k*‐value and C release with SPCs of 0.22 and 0.06 (both *p*‐value < .05), respectively. Soil temperature had a positive effect on *k*‐value and release rates of C, N, and P with SPCs of 0.43 (*p*‐value < .001), 0.31 (*p*‐value < .01), 0.93 (*p*‐value < .001), and 0.59 (*p*‐value < .01), respectively. Soil moisture had a positive effect on *k*‐value with SPC of 0.41 (*p*‐value < .05) and a negative effect on C release with SPC of −0.29 (*p*‐value < .001) (Figure 4a).

Standardized total effects (STE) are shown in Figure 4b. Solar radiation and soil temperature both had higher STE on *k*‐value and release rates of C, N, and P. Aspect had a negative STE on litter decomposition. Plant species diversity had a modest STE on litter decomposition.

## 4 DISCUSSION

In this study, litter mass decomposition and nutrient release occurred at different rates on different terrains. Slope and aspect affect rates of *k*‐value and C, N, and P release and interact with each other in a semiarid area (Table 3). It has been found that temperature, moisture, solar radiation, litter quality, and plant species diversity are important drivers of litter decomposition in drylands (Austin & Vivanco, 2006; Bornman et al., 2015; King et al., 2012). In the current study, the same litter was used for all experimental setting, so litter quality was not a factor. Soil temperature and moisture, solar radiation, and plant species diversity were closely related to the terrains in this study (Table 2). Indeed, soil temperature and solar radiation were the most significant factors affecting the k‐value and C, N, and P release during the decomposition process (Figure 4b). These findings were consistent with those of Poll et al. (2002), who reported that soil temperature was a key driver of decomposition process efficiency as governed by the amount of incident solar radiation.

### 4.1 Contribution of solar radiation and soil temperature to litter decomposition

Many studies have shown that photodegradation can be an important driver of litter decomposition in arid ecosystems (Almagro et al., 2016; King et al., 2012). Austin and Vivanco (2006) reported that photodegradation is a process that breaks down organic matter through solar radiation and directly releases CO_2_, causing the decomposed carbon to be discharged directly into the atmosphere without entering the pool of soil organic matter. Some studies have shown that soil temperature significantly affects litter decomposition (Jenny et al., 2018; Petraglia et al., 2018). Wu et al. (2017) noted that soil warming increases the rate of decomposition. Xiao et al. (2014) found that an increase in soil temperature within the range 0–13°C accelerates litter decomposition, although decomposition slows above 13°C. The soil temperature used in this study was the annual mean temperature ranging from 8.6 to 12.5°C (Table 1), but the soil temperature in the study area can reach 56. 0°C in June (Table S1), and that higher temperature may have slowed litter decomposition. We found that solar radiation and soil temperature were higher on sunny aspect (Table 1), and these two factors were the greatest contributors to litter decomposition and the rates of C, N, and P release (Figure 2b). The greater temperature difference and solar radiation difference between shaded and sunny aspect at steeper slope. So, litter decomposition also shows a big difference at steeper slope (Figure S1). The STE of aspect on litter mass decomposition and C, N, and P release was negative (Figure 3b), That is because the decomposition of litter is not only affected by temperature, but also regulated by soil moisture and the plant species diversity (Xiao et al., 2014). Soil moisture was the most important factor in the rate of litter decomposition (Yoon et al., 2014), but the magnitude of this effect was dependent on soil temperature (Butenschoen et al., 2011). High soil moisture can also potentially cause a greater loss of litter mass (Cotrufo et al., 2015). Petraglia et al. (2018) found that the effect of temperature on the decomposition rate varied with soil moisture. Litter decomposition rate decreased with increasing temperature on the low‐moisture areas and increased with increasing temperature on the high‐moisture areas (Butenschoen et al., 2011). The annual mean soil moisture was lower (ranging from 6.5% to 9.4%; Table 1), which may also explain the negative effect of temperature on the rate of litter decomposition.

### 4.2 Effects of plant species diversity on litter decomposition

The STE of plant species diversity on the litter decomposition and release rates of C, N, and P was positive (Figure 4b) and was consistent with the study of Hector et al. (2000), who found that plant species diversity positively affected litter breakdown. Szanser et al. (2011) also found that the plant species diversity is an important factor for humus accumulation in soil. This is because the increase in plant species diversity leads to an increase in the diversity and amount of decomposers (Tresch et al., 2019), especially those microorganisms responsible for litter decomposition and release of C, indicating that higher plant species diversity can increases the diversity of microorganisms for decomposing mass and C in this study area. The mass loss and C release shown the same trend. This is consistent with the study of Zukswert and Prescott (2017), who found that the amount of C was related to the amount of litter biomass loss. Litaor et al. (2015) found that soil moisture had a positive impact on the surrounding environments. Whereas we found a negative impact on plant species diversity (Figure 2a). Our data are consistent with the study by El‐Ghani et al. (2012), who reported that plant species diversity increases as soil moisture decreases. The dense canopy of tall‐growing species along the water's edge makes germination and growth of other species more difficult, often leading to reduced species diversity (El‐Ghani et al., 2012; Zhang et al., 2018).

### 4.3 Grassland management on different terrains

This study was conducted in a long‐period enclosed grassland (Hu et al., 2019). Long‐term enclosure will result in the accumulation of litter and waste of forage resources (Galvánek & Lep, 2012). Grassland could be used for grazing or mowing. However, the terrain of the study area is mostly slope (Figure 1). Mowing could only be used on the gentle slopes; however, sheep grazing could be used on steep slopes, as sheep can move flexibly on the hillside. In this study, we found that the decomposition rate decreased with increasing slope steepness on shaded aspect, while on sunny aspect, the decomposition rate decreased with decreasing slope steepness (Figure 3). So, sheep grazing could be carried out on shaded steep slopes and mowing could be used on the sunny gentle slopes, both could be used at growth season of vegetation from June to August. Thereby it can reduce the accumulation of litter and promote its decomposition by sheep trampling (Liu et al., 2018).

## 5 CONCLUSIONS

Our results show that early litter mass loss and C, N, and P release were significantly affected by slope, aspect, and their interaction. On shaded aspect, the increase in slope decreased the k‐value, while on sunny aspect, the *k*‐value increased with increasing slope. The *k*‐value of the shaded aspect is faster than that of the sunny aspect at 15° and 30°, while at 45°, the *k*‐value of the sunny aspect is significantly faster than that of the shaded aspect. Soil temperature and solar radiation had the strongest effects of promoting litter mass loss and C, N, and P release, but they depended on soil moisture and plant species diversity. The soil temperature and moisture, solar radiation, and plant species diversity all varied depending on the terrain (aspect and slope). Soil temperature was significantly affected by solar radiation, and the soil moisture could affect the plant species diversity. In sum, terrain influences litter mass loss and C, N, and P release by affecting soil temperature and moisture, solar radiation, and plant species diversity in Loess Plateau, a typical steppe ecoregion. The litter mass loss was slowly on the shaded steep slopes and sunny gentle slopes, and it could be combined with grazing and mowing to reduce the accumulation of litter and accelerate its decomposition.

## CONFLICT OF INTEREST

None declared.

## AUTHOR CONTRIBUTION

**An Hu:** Formal analysis (equal); Writing‐original draft (equal); Writing‐review & editing (equal). **Yaning Duan:** Conceptualization (equal); Data curation (equal). **Lei Xu:** Data curation (equal). **Shenghua Chang:** Data curation (equal). **Xianjiang Chen:** Data curation (equal). **Fujiang Hou:** Conceptualization (equal); Data curation (equal); Writing‐original draft (equal); Writing‐review & editing (equal).

## Supporting information

Supplementary Material

## Data Availability

Data are available through the Figshare (https://doi.org/10.6084/m9.figshare.12110979.v3).
