# Supplementary material for: Litter decomposes slowly on shaded steep slope and sunny gentle slope in a typical steppe ecoregion
Source: Ecol Evol. 2021 Feb 26;11(6):2461–70. doi: 10.1002/ece3.6933 (PMC7981220; doi:10.1002/ece3.6933)
Supplement: Supplementary file 1 — Supplementary Material [file ECE3-11-2461-s001.docx]

Table S1 Dynamics of soil surface temperature in different terrains at 2 pm (°C)

| Aspect |  | Slope |  | 2011 |  |  |  |  |  |  |  |  | 2012 |  |  |
| --- | --- | --- | --- | --- | --- | --- | --- | --- | --- | --- | --- | --- | --- | --- | --- |
|  |  |  |  | Apr | May | Jun | Jul | Aug | Sep | Oct | Nov | Dec | Jan | Feb | Mar |
| Shaded |  | 15° |  | 35.8 | 44.5 | 54.2 | 45.0 | 45.8 | 33.5 | 26.4 | 14.6 | -0.5 | -6.5 | -2.4 | 22.5 |
|  |  | 30° |  | 39.1 | 43.0 | 53.5 | 43.2 | 46.2 | 32.8 | 16.3 | 10.2 | -6.5 | -5.9 | -3.2 | 21.3 |
|  |  | 45° |  | 34.5 | 39.6 | 53.4 | 36.8 | 45.5 | 26.5 | 16.4 | 1.7 | -4.5 | -7.7 | -3.1 | 19.4 |
| Sunny |  | 15° |  | 39.5 | 52.3 | 55.5 | 49.6 | 52.1 | 37.0 | 38.5 | 22.3 | 2.0 | -4.9 | -1.2 | 23.3 |
|  |  | 30° |  | 44.4 | 49.6 | 56.0 | 41.2 | 48.0 | 38.6 | 31.5 | 15.8 | 0.0 | -4.0 | -1.4 | 24.5 |
|  |  | 45° |  | 44.6 | 51.0 | 55.2 | 47.4 | 45.5 | 36.0 | 31.0 | 22.6 | 1.5 | -5.8 | -0.6 | 25.3 |

Figure S1 Relationship between mass loss difference of sunny and shaded aspect and soil temperature difference (a) and solar radiation difference (b) of sunny and shaded aspects in three decomposition periods.
